# Anion Exchange at the Liquid/Solid Interface of Ultrathin Ionic Liquid Films on Ag(111)

**DOI:** 10.1002/cphc.201800773

**Published:** 2018-09-24

**Authors:** Matthias Lexow, Bettina S. J. Heller, Florian Maier, Hans‐Peter Steinrück

**Affiliations:** ^1^ Lehrstuhl für Physikalische Chemie 2 Friedrich-Alexander-Universität Erlangen-Nürnberg Egerlandstr. 3 91058 Erlangen Germany

**Keywords:** ionic liquids, ion pairs, silver, wetting, X-ray photoelectron spectroscopy

## Abstract

Thin ionic liquid (IL) films play an important role in many applications. To obtain a better understanding of the ion distribution within IL mixture films, we sequentially deposited ultrathin layers of two ILs with the same cation but different anions onto Ag(111), and monitored their dynamic behaviour by angle‐resolved X‐ray photoelectron spectroscopy. Upon depositing [C_8_C_1_Im][PF_6_] on top of a wetting layer of [C_8_C_1_Im][Tf_2_N] at room temperature (RT), we found a pronounced enrichment of the [Tf_2_N]^−^ anions at the IL/vacuum interface, due to a rapid anion exchange at the IL/solid interface. In contrast, at 90 K, the [Tf_2_N]^−^ anions remain at the IL/solid interface. Upon heating, we observe a rearrangement of the cations between 140 and 160 K, such that the octyl chains preferentially point towards the vacuum. Above 170 K, the ions start to become mobile, and at 220 K, the anion exchange is completed, with the [Tf_2_N]^−^ anions enriched at the IL/vacuum interface in the same way as found for deposition at RT. The temperature range for the anion exchange corresponds well to glass transition temperatures reported in literature. We propose two driving forces to be cooperatively responsible for the replacement/exchange of [Tf_2_N]^−^ at the IL/solid interface and its enrichment at the IL/vacuum interface. First, the adsorption energy of [C_8_C_1_Im][PF_6_] is significantly larger than that of [C_8_C_1_Im][Tf_2_N], and second, the surface tension of [C_8_C_1_Im][Tf_2_N] is lower than that of [C_8_C_1_Im][PF_6_].

Ionic liquids (ILs) are salts with comparably low melting points, often even below room temperature (RT). As ultrathin films, they are of utmost interest in current and potential future applications in catalysis,^1^, ^2^, ^3^ sensors,^4^ lubrication,^5^,^6^ separation,^7^,^8^ and electrochemistry,^9^ to name only a few. In these examples, the liquid/solid interface determines the function, performance and stability of the respective system. Therefore, and also from a fundamental interest in the structure and formation of liquid/solid interfaces, the adsorption and wetting properties of ILs on solid surfaces are studied on the molecular,^10^,^11^ mesoscopic^12^ and macroscopic scale.^13^ Only detailed knowledge of the interface properties allows for tailoring systems for specific applications. Using mixtures of ILs promises an even larger parameter space for targeted applications of ultrathin IL films. For such systems, many fundamental questions concerning the nature of the IL/support and IL/gas(vacuum) interfaces arise immediately. What is the composition and structure? Are there preferential adsorption, segregation, and enrichment phenomena? How do these effects depend on temperature and on the nature of the IL constituents? All these questions are of high relevance, since the corresponding behaviour determines the nature and stability of these interfaces in any technical application.

The extremely low vapour pressure and excellent thermal stability of many ILs enable not only their use under extreme conditions, but also their investigation in ultra‐high vacuum (UHV) under well‐defined conditions with atomic level accuracy.^2^,^14^,^15^ Physical vapour deposition (PVD) combined with angle‐resolved X‐ray photoelectron spectroscopy (ARXPS) has proven as a well‐established method to investigate IL/solid interactions, wetting behaviour, and IL film growth.^16^ The accessible coverages range from sub‐monolayers to several multilayers.^16^, ^17^, ^18^, ^19^, ^20^ In previous model studies, the properties of the liquid/solid interface of ultrathin IL films were found to depend on the structure of the IL,^17^ the nature of the solid,^21^ and temperature.^10^,^18^,^22^

Herein, we investigate phenomena that occur when ultrathin layers of ILs are successively deposited on the Ag(111) surface. Using ARXPS, we demonstrate that ion exchange processes occur and how they can be influenced by temperature. The two investigated ILs are 1‐methyl‐3‐octyl imidazolium bis[(trifluoromethyl)sulfonyl]imide, [C_8_C_1_Im][Tf_2_N], and 1‐methyl‐3‐octyl imidazolium hexafluorophosphate, [C_8_C_1_Im][PF_6_]. While the former has been studied as neat IL on Ag(111) before,^10^,^15^ making this system an excellent reference system, the latter is so far unexplored in this context. The different chemical environments in the two anions (top of Figure 1) yield distinct chemical shifts of the respective F 1s binding energies in XPS. This allows for a direct comparison of the relative occurrence of the respective anion in the bulk and at the interfaces of ultrathin IL films. Changes in signal intensity by varying surface sensitivity in ARXPS directly reflect surface enrichment or depletion of one of the anions.

**Figure 1:**
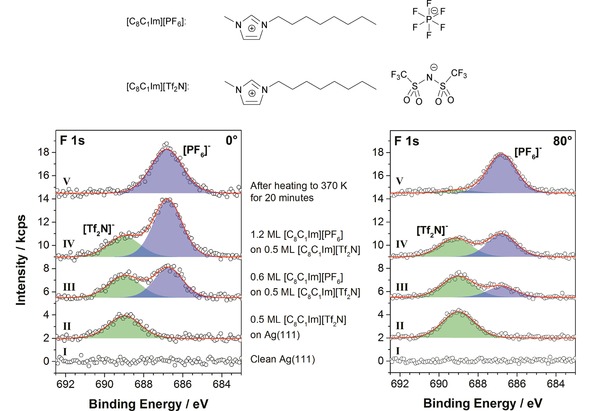
F 1s region measured in 0° (left) and 80° (right) emission for the clean Ag(111) crystal (I), after deposition of 0.5 ML of [C_8_C_1_Im][Tf_2_N] (II), after two subsequent deposition steps of 0.6 ML of [C_8_C_1_Im][PF_6_] on top of the existing [C_8_C_1_Im][Tf_2_N] layer (III and IV), and after heating the layered system to 370 K for 20 min (V). All spectra have been measured at RT. At the top of the figure, the molecular structures of 1‐methyl‐3‐octylimidazolium hexafluorophosphate, [C_8_C_1_Im][PF_6_], and 1‐methyl‐3‐octylimidazolium bis[(trifluoromethyl)sulfonyl]imide, [C_8_C_1_Im][Tf_2_N] are shown.

To begin with, we discuss the effects occurring upon successive deposition of [C_8_C_1_Im][Tf_2_N] and [C_8_C_1_Im][PF_6_] at room temperature (RT). Figure 1 shows F 1s ARXP spectra at emission angles of 0° and 80°, respectively. Measurements at 0° (normal emission) probe the near‐surface region with an information depth (ID) of 7–9 nm (depending on the kinetic energy of the electrons), measurements at 80° (grazing emission) only the topmost surface layers with an ID of 1–1.5 nm. ID is defined as 3 times the inelastic mean free path *λ* of an electron at a given kinetic energy. IL coverages are given in ML, where 1 ML is defined as a closed double layer of ions irrespective of their relative arrangement.^16^,^17^ Based on the ILs’ molecular volume $V_{m}$ , the height h of 1 ML was estimated to be 0.77 nm for [C_8_C_1_Im][PF_6_] and 0.84 nm for [C_8_C_1_Im][Tf_2_N], using values for $V_{m}$ from literature.^10^,^17^,^23^

As a first step, an equivalent of 0.5 ML [C_8_C_1_Im][Tf_2_N] was deposited (Figure 1‐II). It was shown previously that this amount forms a homogeneous wetting layer (WL) on Ag(111), with the IL ions arranged in a so‐called checkerboard arrangement.^10^ In this layer, the anions are adsorbed in a *cis*‐conformation, with the CF_3_ groups preferentially pointing towards the vacuum, as depicted schematically in Figure 2‐II. This orientation results in a relative enhancement of the corresponding XP signals in the surface sensitive geometry, that is, at 80°.^10^

**Figure 2:**
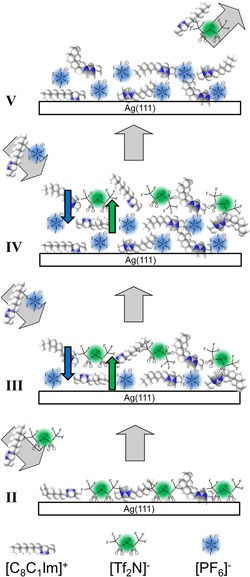
Scheme of the ion exchange process during step‐wise deposition of [C_8_C_1_Im][PF_6_] onto a WL of [C_8_C_1_Im][Tf_2_N] on Ag(111) at RT and the selective desorption of [C_8_C_1_Im][Tf_2_N] at 370 K. The Roman numbers refer to the spectra in Figure 1.

As a second step, we deposited an equivalent of 0.6 ML [C_8_C_1_Im][PF_6_] on top of the [C_8_C_1_Im][Tf_2_N] WL at RT (Figure 1‐III); note that this amount of [C_8_C_1_Im][PF_6_] also forms a closed WL on clean Ag(111) (see SI, Figure S3). The corresponding F 1s spectrum in the bulk‐sensitive geometry at 0° (where damping effects are negligible for such thin layers) shows the expected intensity ratio $F_{{\mathrm{Tf}}_{2}N}/F_{{\mathrm{PF}}_{6}}$ of 0.8 for the anions (nominal value: 0.5 ML/0.6 ML=0.83). In the surface‐sensitive geometry at 80°, however, a ratio $F_{{\mathrm{Tf}}_{2}N}/F_{{\mathrm{PF}}_{6}}$ of 2.2 is found. This very large value indicates a strong enrichment of the [Tf_2_N]^−^ anions at the IL/vacuum interface. Given the fact that prior to the deposition of [C_8_C_1_Im][PF_6_] the [Tf_2_N]^−^ anions were in direct contact with the underlying Ag surface, this can only be explained by a rapid exchange of the anions at the IL/Ag interface, as is schematically indicated in Figure 2‐III. The spectrum of the composite layer was acquired 3 minutes after the deposition has ended and no changes were observed even after one hour. Hence, the ion exchange had already occurred on a much shorter time‐scale, before the first spectra acquisition was started.

As a third step, we deposited an additional 0.6 ML of [C_8_C_1_Im][PF_6_] on top of the previous composite IL film, yielding a total [C_8_C_1_Im][PF_6_] coverage of 1.2 ML (Figure 1‐IV). The 0° F 1s spectrum shows the expected intensity ratio $F_{{\mathrm{Tf}}_{2}N}/F_{{\mathrm{PF}}_{6}}$ of 0.4 (nominal value: 0.5 ML/1.2 ML=0.42). At 80°, the larger ratio of $F_{{\mathrm{Tf}}_{2}N}/F_{{\mathrm{PF}}_{6}}$ =0.9 again indicates a relative enrichment of the [Tf_2_N]^−^ anions at the IL/vacuum interface, as schematically shown in Figure 2‐IV.

To investigate its thermal stability, we heated the film after the third deposition step for 20 minutes at 370 K (Figure 1‐V), which led to the complete disappearance of the [Tf_2_N]^−^ signal. We attribute this observation to selective desorption of [C_8_C_1_Im][Tf_2_N], Figure 2‐V. Notably, the pure WL of [C_8_C_1_Im][Tf_2_N] on Ag(111), i. e. a film of 0.5 ML thickness (Figure 1‐II) directly in contact with the surface, is stable up to 410 K. This is evident from Figure 3, where the thermal evolution of pure [C_8_C_1_Im][Tf_2_N] and [C_8_C_1_Im][PF_6_] films with initial coverages of ∼1.5 ML are compared. Clearly, the successive desorption of multilayers and the wetting layers is observed for the individual ILs. The lower multilayer desorption temperature of [C_8_C_1_Im][Tf_2_N] can be explained by the considerably lower enthalpy of vapourisation Δ_vap_ H_298K_ of 150 kJ/mol as compared to 169 kJ/mol for [C_8_C_1_Im][PF_6_].^24^ It is also in line with the absolute vapour pressures of both ILs, which were determined at slightly higher temperatures of 423 K by Verevkin and co‐workers, yielding ∼400 μPa for [C_8_C_1_Im][Tf_2_N] and ∼8 μPa of [C_8_C_1_Im][PF_6_].^25^ In case of the mixed IL film on Ag(111), the complete disappearance of the F 1s signal of [C_8_C_1_Im][Tf_2_N] at 370 K in Figure 1‐V thus clearly shows that the WL of [C_8_C_1_Im][Tf_2_N] is destabilised by postdeposition of [C_8_C_1_Im][PF_6_] and desorbs at a temperature close to that of [C_8_C_1_Im][Tf_2_N] multilayer desorption (Figure 3).

**Figure 3:**
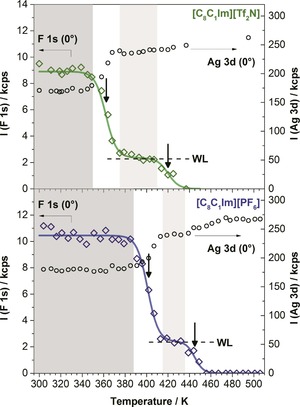
Intensity of F 1s and Ag 3d signals from XPS in 0° emission as a function of temperature, upon continuous heating of thin films of [C_8_C_1_Im][Tf_2_N] (1.6 ML) and [C_8_C_1_Im][PF_6_] (1.4 ML) from RT to 500 K with a heating rate of 2 K/min. The stability range of the multilayers is shaded in dark grey, that of the isolated WLs in light grey. The desorption temperatures of the multilayers and wetting layers, that is, the rate maxima (as determined from the inflection points of the decreasing signals), are indicated as vertical arrows. For [C_8_C_1_Im][Tf_2_N], the multilayer and WL desorption temperatures are 365 and 420 K, respectively, and for [C_8_C_1_Im][PF_6_] 405 and 445 K, respectively.

To study the dynamics of the ion‐exchange process, we deposited thin IL films on Ag(111) at a much lower temperature of 90 K; see Figures 4 and 5. Again, first ∼0.6 ML of [C_8_C_1_Im][Tf_2_N], the equivalent of the WL, were deposited. According to STM studies, several crystalline and amorphous condensed phases are formed in the WL at this low temperature, all with an overall checkerboard arrangement of alternating anions and cations.^15^ The observed *cis*‐orientation of the [Tf_2_N]^−^ anion at low temperature with the CF_3_‐groups preferentially pointing towards the vacuum^15^ is confirmed here (Figure 4‐II) from the intensity ratios of the C 1s signals (C_Tf2N_ denotes the two carbon atoms in the CF_3_ groups of the anion, C_hetero_ the five carbon atoms bound to the nitrogen atoms of the imidazolium ring, and C_alkyl_ the remaining seven alkyl carbon atoms^23^). At 80°, the C_Tf2N_/C_hetero_ ratio of 0.6 is larger than the nominal ratio of 0.40 (=2/5), which indicates a relative enrichment of the CF_3_ groups of the [Tf_2_N]^−^ anion at the vacuum interface. This preferential anion orientation in the WL was also observed in XPS at RT.^10^ Information on the orientation of the octyl chain of the [C_8_C_1_Im]^+^ cation is obtained from the C_hetero_/C_alkyl_ ratio at 80°. The value of 0.6 at 90 K is close to the nominal ratio of 0.71 (=5/7). It has to be compared to the much lower value of ∼0.4 observed for the same film thickness at RT.^10^ The latter is attributed to the pronounced preferential orientation of the alkyl chains towards the vacuum at RT, which leads to an attenuation of the signal from the imidazolium rings underneath.^10^ The ratio of 0.6 observed at 90 K indicates a much less pronounced preferential orientation of the alkyl chains towards the vacuum in the frozen state. This likely is due to a hit‐and‐stick adsorption mechanism at this low temperature.

**Figure 4:**
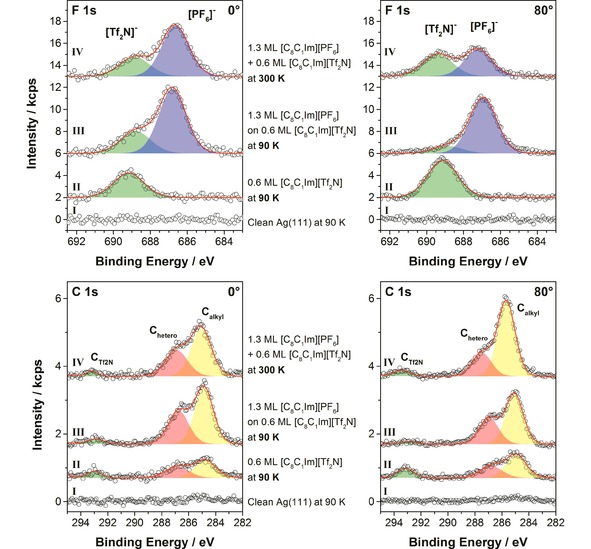
F 1s and C 1s spectra in 0° and 80° emission for the clean Ag(111) crystal (I), after deposition of 0.6 ML of [C_8_C_1_Im][Tf_2_N] at 90 K (II), after deposition of 1.3 ML of [C_8_C_1_Im][PF_6_] on top of the existing film of [C_8_C_1_Im][Tf_2_N] at 90 K (III), and after heating the composite IL film to 300 K (IV).

In a second step, 1.3 ML of [C_8_C_1_Im][PF_6_] were deposited on top of the [C_8_C_1_Im][Tf_2_N] WL of 0.6 ML thickness at 90 K (Figure 4‐III). The strong attenuation of the F_Tf2N_ and C_Tf2N_ signals at 80° clearly shows that the deposited [C_8_C_1_Im][PF_6_] homogeneously covers the underlying [C_8_C_1_Im][Tf_2_N] layer, and indicates that no exchange of anions occurred at this low temperature. The situation is sketched in Figure 5‐III.

**Figure 5:**
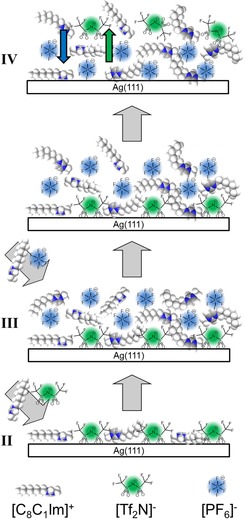
Scheme for the heating experiment after the deposition of [C_8_C_1_Im][PF_6_] to a WL of [C_8_C_1_Im][Tf_2_N] on Ag(111) at 90 K. The Roman numbers refer to the spectra in Figure 4.

Next, we heated this layered IL film step‐wise to RT, and measured XP spectra *in situ* at each temperature. The final spectrum at RT is shown in Figure 4‐IV, and the individual F 1s spectra acquired at 80° during heating are depicted in Figure S6 in the SI. Figure 6 shows the corresponding quantitative analysis. Initially, there is no notable change in the [PF_6_]^−^ and [Tf_2_N]^−^ peak intensities. Starting at 140 K, the [PF_6_]^−^ signal (blue) and also the total sum (black) decreases while the [Tf_2_N]^−^ signal (green) remains constant up to 170 K. We attribute this behaviour to a reorganisation within the uppermost layer in this temperature window, such that the octyl chains are enriched at the IL/vacuum interface, that is, they point towards the vacuum, thereby attenuating the [PF_6_]^−^ signal at 80°. The [Tf_2_N]^−^ anions are not affected by this reorientation at the outer surface and remain at the IL/Ag interface, indicating that this temperature is still too low for diffusion/ion exchange to occur. Above 170 K, the [Tf_2_N]^−^ signal begins to increase and simultaneously the [PF_6_]^−^ signal continues to decrease. At this temperature, the exchange of the anions in contact with the Ag surface starts. The [Tf_2_N]^−^ enrichment continues to increase until 220 K, and thereafter remains constant up to RT, indicating that no further changes occur at the IL/vacuum interface (Figure 5‐IV). As is to be expected, the 80° spectra after heating to RT have the same spectral shape as the spectra after deposition at RT (compare Figure 4‐IV to Figure 1‐IV). The temperature range for the anion exchange corresponds well to the glass transition temperatures of bulk [C_8_C_1_Im][Tf_2_N] and [C_8_C_1_Im][PF_6_], which were reported to be around 185 K^26^, ^27^, ^28^ and 190 K,^29^,^30^ respectively. In agreement to the bulk glass transition, Uhl *et al*. observed in STM that for coverages below 0.5 ML ordered structures in films of [C_8_C_1_Im][Tf_2_N] on Ag(111) melted around 185 K.^15^

**Figure 6:**
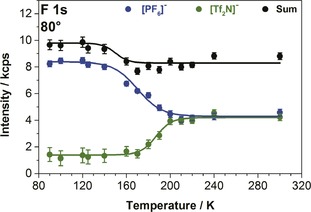
Intensities of the respective F 1s signals of the [PF_6_]^−^ and [Tf_2_N]^−^ anions from XPS in 80° emission as a function of the sample temperature upon heating from 90 to 300 K (selected spectra are shown in SI, Figure S6).

Interestingly, the comparison of ultrathin [C_8_C_1_Im][Tf_2_N]/[C_8_C_1_Im][PF_6_] films with macroscopically thick films (0.1 mm) of the same stoichiometry shows an identical IL/vacuum interface composition at RT (see Figures S4 and S5 in the SI), that is, an enrichment of the [Tf_2_N]^−^ anion.

In conclusion, we discovered ion exchange processes in ultrathin films of the ionic liquids [C_8_C_1_Im][Tf_2_N] and [C_8_C_1_Im][PF_6_], which were subsequently deposited on Ag(111), and studied the dynamics by quantitative angle‐resolved XPS. Both ILs form wetting layers, with anions and cations in direct contact with the metal, arranged in a checkerboard structure. Upon depositing [C_8_C_1_Im][PF_6_] on top of the [C_8_C_1_Im][Tf_2_N] wetting layer at RT, we find a pronounced enrichment of the [Tf_2_N]^−^ anions at the IL/vacuum interface. This effect is explained by a rapid anion exchange at the IL/solid interface. In contrast, after deposition of [C_8_C_1_Im][PF_6_] at 90 K the [Tf_2_N]^−^ anions remain at the IL/solid interface. Upon heating, the arrangement of the ions within the thin IL film changes. Between 140 and 160 K, we observe a rearrangement of the cations, such that the octyl chains point towards the vacuum. Above 170 K, the ions start to become mobile, and at 220 K, the anion exchange is completed, with the [Tf_2_N]^−^ anions enriched at the IL/vacuum interface, as was observed for deposition at RT. The temperature range of the anion exchange corresponds well to glass transition temperatures reported in literature. By subsequently heating to 370 K, it is possible to selectively desorb [C_8_C_1_Im][Tf_2_N] already at a temperature, which is much lower than the temperature of >420 K required for desorption of the neat WL. This shows that the WL of [C_8_C_1_Im][Tf_2_N] can be destabilised by postdeposition of [C_8_C_1_Im][PF_6_]. This opens new routes for selectively removing specific ions or undesired components at IL/support interfaces.

We propose two driving forces to be cooperatively responsible for the replacement/exchange of [Tf_2_N]^−^ at the IL/solid interface and its enrichment at the IL/vacuum interface. First, the adsorption energy of [C_8_C_1_Im][PF_6_] is significantly larger than that of [C_8_C_1_Im][Tf_2_N], as deduced from the desorption temperatures of the neat WLs, that is, 445 vs 420 K, respectively (see arrows in Figure 3). Second, the enrichment of [Tf_2_N]^−^ at the IL/vacuum interface, which is observed for ultrathin films and also for thick films, where the influence of the IL/Ag interface is negligible, is likely related to the lower surface tension of [C_8_C_1_Im][Tf_2_N], 29 mN/m, as compared to 32 mN/m for [C_8_C_1_Im][PF_6_].^23^ Our results highlight how the interface compositions of thin multi‐component IL films depend on the nature of the ions, and how they can be controlled via temperature. They add to the understanding of IL/solid and IL/vacuum interfaces, which play a crucial role in IL thin film applications for lubrication, coating, separation, electrochemistry, sensor, and catalysis technologies.

## **Experimental Section**

The ILs were deposited onto a circular Ag(111) single crystal (15 mm diameter, 2 mm thickness) purchased from MaTecK with a purity of 99.999 % and one side polished to the (111) plane with an accuracy better than 0.1°. The crystal was mounted to a Mo sample holder with transferable electric contacts and fixed with Ta wires.^10^ The crystal temperature was measured with an absolute accuracy of ±20 K and a reproducibility of ±2 K, using a type K thermocouple put into a 0.5 mm pinhole of the crystal. Surface preparation was done in UHV by repetitive cycles of sputtering with 0.6 keV Ar^+^ ions (8 μA, 30 min), followed by annealing at 800 K. The final sample cleanliness and long range order were checked by XPS and low energy electron diffraction (LEED), respectively. [C_8_C_1_Im][PF_6_] was purchased from Sigma‐Aldrich (purity >95 %). [C_8_C_1_Im][Tf_2_N] was synthesised under ultrapure conditions according to previous publications.^31^ For comparing the thin film results with bulk mixtures, bulk amounts of [C_8_C_1_Im][Tf_2_N]/[C_8_C_1_Im][PF_6_] mixtures were prepared with various compositions using acetonitrile (Sigma‐Aldrich, purity 99.8 %) as a co‐solvent to ensure proper mixing of the respective ILs;^32^ the IL mixture was spread as macroscopic film (about 0.1 mm thickness) on polycrystalline Ag foil. After careful degassing of the liquid film, the sample was introduced into the vacuum chamber for ARXPS.

The UHV system used for this study has been described previously for the deposition of [C_8_C_1_Im][Tf_2_N] on Ag(111).^10^ For this sequential film deposition study, well‐defined amounts of the two different ILs were deposited onto the freshly prepared Ag(111) crystal via PVD using two Knudsen cells, developed in our group specifically for IL deposition (see Figures S1 and S2). Both ILs were carefully degassed in UHV for more than 24 hours at evaporator temperatures between 370 and 430 K to remove volatile impurities. The crucible temperatures during evaporation ranged from 428 to 443 K for [C_8_C_1_Im][PF_6_], and from 403 to 413 K for [C_8_C_1_Im][Tf_2_N]. The higher temperatures for [C_8_C_1_Im][PF_6_] are due to its higher enthalpy of vapourisation.^10^,^24^ The maximum chamber background pressures during the deposition were below 3×10^−9^ mbar for [C_8_C_1_Im][PF_6_] and below 8×10^−10^ mbar for [C_8_C_1_Im][Tf_2_N]. At the applied temperatures, the ILs arrived on the target surface as single ion pairs without any signs of decomposition, in line with literature.^10^,^16^,^22^,^24^ This behaviour was confirmed by comparing the XP spectra of our deposited films to spectra of macroscopically thick IL films prepared *ex‐situ* (see Table S1). The IL flux during PVD was checked using a quartz crystal microbalance (QCM) for each deposition experiment in order to verify stable evaporation rates.

Angle‐resolved XP spectra were acquired with a VG SCIENTA R3000 hemispherical electron analyser at polar emission angles of *ϑ*=0° and 80° with respect to the surface normal. The non‐monochromated SPECS XR 50 Al Kα X‐ray source (1486.6 eV photon energy) was operated at a power of 240 W. All spectra were measured with a pass energy of 100 eV yielding an overall energy resolution of about 0.9 eV.

Background subtraction and peak fitting was done using CasaXPS V2.3.16Dev6. The background of the Ag 3d and F 1s core levels were subtracted applying the Shirley method.^33^ O 1s, P 2p, and S 2p core levels were treated using a two‐point linear background, C 1s with a three‐point linear background. In the N 1s region, the overlap with plasmons, shake‐up satellites, and the inelastically scattered electrons of the Ag 3d lines made the subtraction of an additional background necessary.^34^, ^35^, ^36^, ^37^ For further details, see reference.^10^ The IL spectra were fitted with a Voigt profile (30 % Lorentzian contribution). For the C 1s spectra, a constraint was applied for the full width at half maximum, fwhm(C_hetero_)= 1.11×fwhm(C_alkyl_), in accordance with previous studies.^10^,^17^,^31^ All binding energies reported were referenced to the Ag Fermi edge, yielding a BE of 368.2 eV for the Ag 3d_5/2_ level.

ARXPS measurements at normal emission, i. e., at *ϑ=*0°, probe the near‐surface region with an information depth, ID, of 7–9 nm (depending on the kinetic energy); measurements at *ϑ*=80°, probe the topmost surface layers with an ID of 1–1.5 nm. ID is defined as three times the inelastic mean free path, *λ*, of an electron at a given kinetic energy. We characterised the growth of the IL films on Ag(111) by measuring the IL signals and the attenuation of the Ag 3d substrate signal as a function of the amount of deposited IL. For homogeneous two‐dimensional IL growth, the Ag 3d intensity at the angle *ϑ* decreases from its maximum intensity *I_0_* for the clean surface to a value *I_d_* for a film with thickness *d*:^17^,^21^

(1) $$\frac{I_{d}}{I_{0}}=e^{-}\frac{d}{\lambda \cdot cos\vartheta }$$

For Ag 3d electrons with a kinetic energy around 1.1 keV, *λ* is 2.5 nm.^10^

For the temperature‐dependent XPS measurements, the sample was heated step‐wise and kept isothermal during the measurement at the respective temperature. The acquisition time for the F 1s region ranged from two to five minutes.

## Conflict of interest

The authors declare no conflict of interest.

## Supporting information

As a service to our authors and readers, this journal provides supporting information supplied by the authors. Such materials are peer reviewed and may be re‐organized for online delivery, but are not copy‐edited or typeset. Technical support issues arising from supporting information (other than missing files) should be addressed to the authors.

Supplementary
