# Supplementary material for: Anion Exchange at the Liquid/Solid Interface of Ultrathin Ionic Liquid Films on Ag(111)
Source: Chemphyschem. 2018 Sep 24;19(22):2978–84. doi: 10.1002/cphc.201800773 (PMC6282575; doi:10.1002/cphc.201800773)
Supplement: Supplementary file 1 — Supplementary [file CPHC-19-2978-s001.pdf]

Supporting Information

© Copyright Wiley-VCH Verlag GmbH & Co. KGaA, 69451 Weinheim, 2018

Anion Exchange at the Liquid/Solid Interface of Ultrathin Ionic Liquid Films on Ag(111)

Matthias Lexow, Bettina S. J. Heller, Florian Maier, and Hans-Peter Steinrück*© 2018 The Authors. Published by Wiley-VCH Verlag GmbH & Co. KGaA.
This is an open access article under the terms of the Creative Commons Attribution License, which permits use, distribution and reproduction in any medium, provided the original work is properly cited.

Ionic Liquid Evaporator

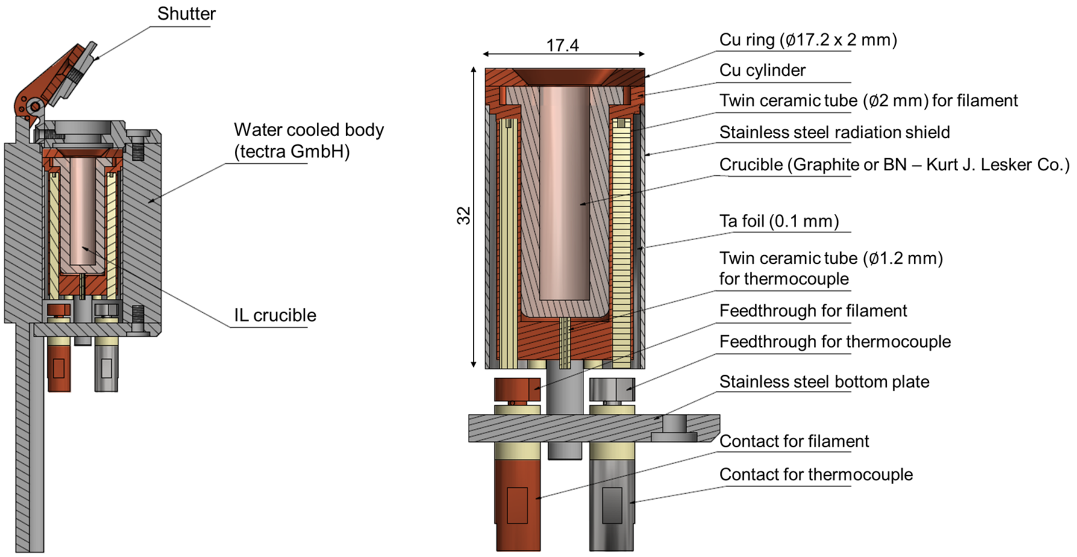

Figure S1: Sketch of the effusion cell developed by our group specifically for the evaporation of ILs in UHV. The water cooled body with shutter was acquired from tectra GmbH. A crucible (purchased from Kurt J. Lesker Co.) sits in a copper cylinder which is heated by a tantalum filament (Ø = 0.25 mm). This filament winds through six ceramic twin tubes (Ø = 2 mm) which are distributed equidistantly in the wall bores of the copper cylinder. A copper ring holds the crucible in place. A type K thermocouple (Ø = 0.125 mm) is contacted to the bottom of the crucible to ensure a stable and accurate temperature reading. Note that the wall bores for the filaments do not go all the way through. In this way, the setup quite effectively prevents ILs (which tend to creep out of the crucible) from direct contact with the hot filaments, which would cause decomposition of the ions and possible contaminations on the target. Figure S2 shows photographs of the setup during assembly.

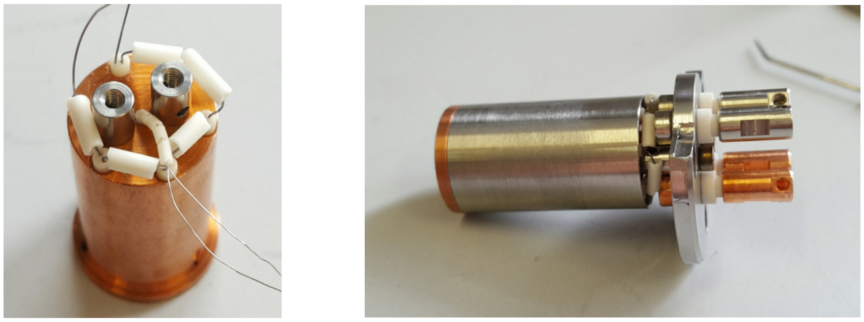

Figure S2: Photographs of the effusion cell during assembly.

Table S1: Film composition as measured by XPS of a 4 nm thick film of $[C_8C_1Im][PF_6]$ on Ag(111) from PVD (left) and a macroscopic droplet (right, italics) at RT in normal emission (0°, top) and grazing emission (80°, bottom). The nominal ratios are given in brackets.

**0°**	C_{alkyl} 1s	C_{hetero} 1s	N 1s	P 2p	F 1s
**atom ratio**	**7.0 / *7.2* (7)**	**5.0 / *5.1* (5)**	**1.8 / *1.9* (2)**	**1.0 / *1.0* (1)**	**6.3 / *5.7* (6)**
FWHM / eV	1.6 / *1.4*	1.7 / *1.8*	1.3 / *1.4*	1.1 / *1.3*	1.7 / *1.7*
BE / eV	285.8 / *285.0*	287.7 / *286.9*	402.9 / *402.1*	137.4 / *136.6*	687.4 / *686.8*

**80°**	C_{alkyl} 1s	C_{hetero} 1s	N 1s	P 2p	F 1s
**atom ratio**	**10.8 / *10.0* (7)**	**4.2 / *4.4* (5)**	**1.4 / *1.5* (2)**	**1.0 / *1.0* (1)**	**3.6 / *4.1* (6)**
FWHM / eV	1.5 / *1.4*	1.7 / *1.6*	1.5 / *1.5*	1.4 / *1.2*	1.7 / *1.7*
BE / eV	286.0 / *285.0*	287.8 / *286.7*	402.9 / *401.8*	137.4 / *136.6*	687.5 / *686.4*

Growth of $[C_8C_1Im][PF_6]$ on Ag(111)

In contrast to $[C_8C_1Im][Tf_2N]$,[1] ultrathin $[C_8C_1Im][PF_6]$ films deposited on Ag(111) were not studied before. Figure S3 shows the attenuation of the Ag 3d substrate signal I_d/I_0 as a function of the IL film thickness after deposition of $[C_8C_1Im][PF_6]$ on Ag(111) for 0° and 80° emission. As expected, the substrate signal intensity decreases with increasing film thickness. Up to 0.5 ML coverage, the measured intensity ratios I_d/I_0 for 0° and 80° strictly coincide with the calculated values (solid and dashed lines, respectively), indicating the formation of a 2D WL on Ag(111) with a checkerboard arrangement of alternating anions and cations. A similar interface structure was observed in previous studies for the growth of $[C_8C_1Im][Tf_2N]$ on Ag(111) by ARXPS at RT[1] and by STM at 110 K.[2]

Upon further deposition on Ag(111), we find that the values for 80° are systematically larger than the calculated curve, indicating a certain degree of 3D morphology. A virtually identical attenuation behaviour of the Ag 3d signal in ARXPS, and thus, a very similar growth on top of a closed WL, was also observed by ARXPS for $[C_8C_1Im][Tf_2N]$ on Ag(111).[1] Since overall small deviations from the curve for ideal 2D growth are found for both ILs, the films finally obtained can be considered to be relatively flat.

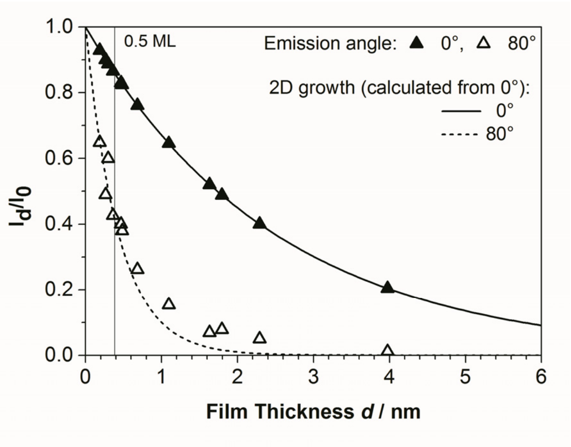

Figure S3: Attenuation of the Ag 3d intensity in 0° and 80° as a function of the layer thickness d of the IL $[C_8C_1Im][PF_6]$ on Ag(111) at RT. The curves for 2D growth are calculated from Equation (1) with an inelastic mean free path λ of 2.5 nm for the corresponding detection angles.

Macroscopic reference mixtures

Figures S4 and S5 show the results from XPS in 80° emission for the surface composition of mixed thin films from PVD and bulk mixtures. This comparison shows that the surface composition is, within the margin of error, independent of the film thickness.

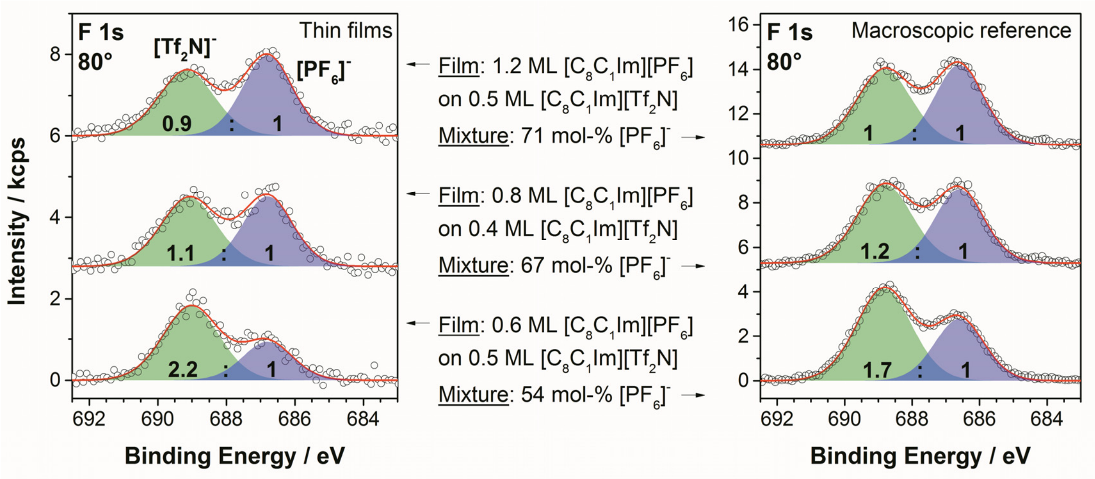

Figure S4: Surface composition at RT as measured by XPS in 80° emission of thin films from PVD (left) compared to macroscopic IL mixtures prepared *ex-situ* (right).

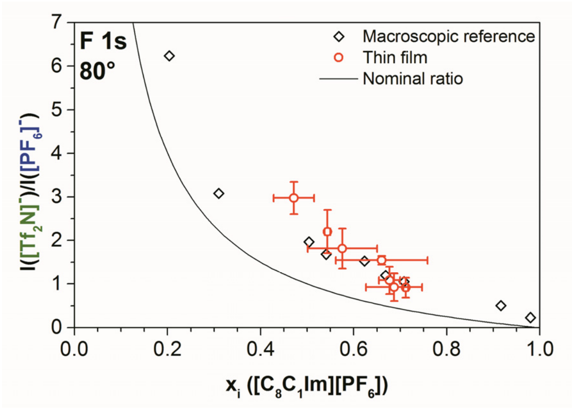

Figure S5: Anion ratio at the surface deduced from F 1s XP spectra in 80° emission for IL mixtures of varying composition. The thickness of the thin films from PVD varied between 0.8 and 1.6 nm.

Heating of layered IL films from 90 to 300 K

Figure S6 shows the individual F 1s XP spectra acquired from XPS in 80° emission during step-wise heating of the layered IL film after deposition of 1.3 ML of $[C_8C_1Im][PF_6]$ on top of a previously deposited existing film of 0.6 ML of $[C_8C_1Im][Tf_2N]$ at 90 K. Figure 6 shows the corresponding quantitative analysis for the F 1s core levels.

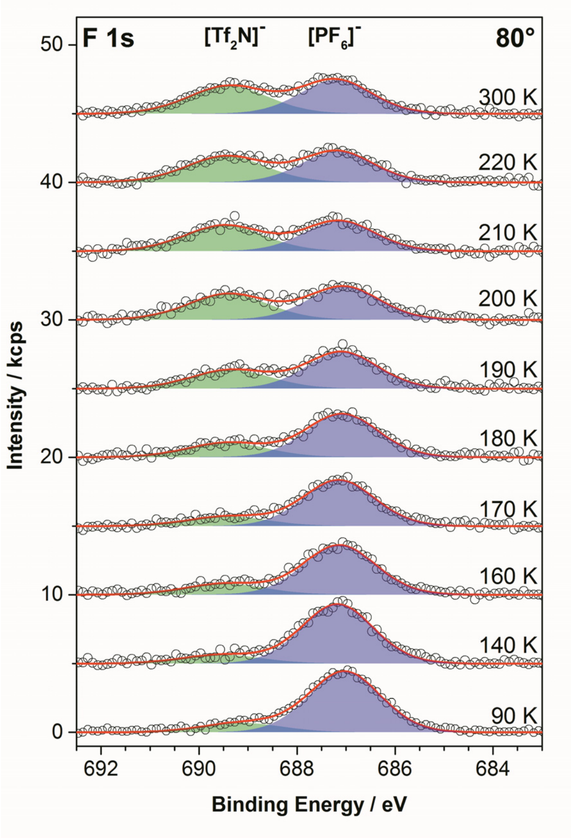

Figure S6: Series of F 1s XP spectra at 80° emission angle upon step-wise heating of the layered IL film after deposition of 1.3 ML of $[C_8C_1Im][PF_6]$ on top of a previously deposited film of 0.6 ML of $[C_8C_1Im][Tf_2N]$ at 90 K., cf. Figure 6.

References

[1] M. Lexow, T. Talwar, B. S. J. Heller, B. May, R. G. Bhuin, F. Maier, H.-P. Steinrück *Phys. Chem. Chem. Phys*. **2018**, 20, 12929-12938.

[2] B. Uhl, H. Huang, D. Alwast, F. Buchner, R. J. Behm *Phys. Chem. Chem. Phys*. **2015**, 17, 23816-23832.
